# Gender inequities in curative and preventive health care use among infants in Bihar, India

**DOI:** 10.7189/jogh.07.020402

**Published:** 2017-12

**Authors:** Rohan J Vilms, Lotus McDougal, Yamini Atmavilas, Katherine Hay, Daniel P Triplett, Jay Silverman, Anita Raj

**Affiliations:** 1 Department of Pediatrics, Duke University Medical Center, Durham, North Carolina, USA; 2 Center on Gender Equity and Health, Department of Medicine, University of California, San Diego School of Medicine, San Diego, California, USA; 3 Bill and Melinda Gates Foundation, New Delhi, India

## Abstract

**Background:**

India has the highest rate of excess female infant deaths in the world. Studies with decade-old data suggest gender inequities in infant health care seeking, but little new large-scale research has examined this issue. We assessed differences in health care utilization by sex of the child, using 2014 data for Bihar, India.

**Methods:**

This was a cross-sectional analysis of statewide representative survey data collected for a non-blinded maternal and child health evaluation study. Participants included mothers of living singleton infants (n = 11 570). Sex was the main exposure. Outcomes included neonatal illness, care seeking for neonatal illness, hospitalization, facility-based postnatal visits, immunizations, and postnatal home visits by frontline workers. Analyses were conducted via multiple logistic regression with survey weights.

**Findings:**

The estimated infant sex ratio was 863 females per 1000 males. Females had lower rates of reported neonatal illness (odds ratio (OR) = 0.7, 95% confidence interval (CI) = 0.6–0.9) and hospitalization during infancy (OR = 0.4, 95% CI = 0.3–0.6). Girl neonates had a significantly lower odds of receiving care if ill (80.6% vs 89.1%; OR = 0.5; 95% CI = 0.3–0.8) and lower odds of having a postnatal checkup visit within one month of birth (5.4% vs 7.3%; OR = 0.7, 95% CI = 0.6–0.9). The gender inequity in care seeking was more profound at lower wealth and higher numbers of siblings. Gender differences in immunization and frontline worker visits were not seen.

**Interpretation:**

Girls in Bihar have lower odds than boys of receiving facility–based curative and preventive care, and this inequity may partially explain the persistent sex ratio imbalance and excess female mortality. Frontline worker home visits may offer a means of helping better support care for girls.

Gender inequities, or the lesser treatment of and opportunities for women and girls relative to men and boys, compromise maternal and child health globally [1–3]. Such inequities are well–documented in India [4–7], which has an overall sex ratio of 919 females per 1000 males [4] Although much of the sex ratio imbalance in India has been attributed to sex–selective abortion [1,8], the country also has the highest rate of excess female infant and child mortality in the world, with 8.8 more female infants dying per 1000 live births than predicted based on global estimates [9]. Gender differences in health care utilization, particularly in the critical first year of life, may be driving this excess mortality for girls in India [10–12].

Gender differences in child health care utilization in India have been documented in multiple forms of health care use [12], but have been largely understood from studies of immunization using nationally representative household survey data from India from 1992 to 2006. This work consistently documents significantly lower rates of immunization for girls in both India and Bihar [13–15]. Further, these studies find that the disparity was enhanced for poorer mothers and based on number of siblings, where full immunization was most likely for oldest males and males born after daughters and least likely for girls with older siblings of either sex [15–17]. Although less research has been conducted on other health care utilization in India, that which has been done documents gender inequities in care seeking for infants and children ill with diarrhea, respiratory or other infectious disease [12,18–20], as well as hospital visits and discharge against medical advice [21–23]. Studies from India document that parents provide up to four times the household expenditure for male relative to female infants, due to both non–use of care and use of less costly care for girls (ie, relying on public rather than preferred private providers) [18,24,25]. Much of this research relies on population–based data more than a decade old or reports from individual health centers. As such, population–based research is needed to examine the current state of gender inequity in infant and child health care use. A trajectory of improvement in gender inequity in immunizations was seen in the period of 1992 to 2006 [17], underscoring the need for more recent reports as expansion of immunization and other health programs has proceeded.

Home visits by frontline workers (FLWs) – community health workers (Accredited Social Health Activists or ASHAs), auxiliary nurse midwives, and social welfare workers for children (Anganwadis) – extend public health care reach to marginalized women in low resource settings [26], and have demonstrated effectiveness and cost–effectiveness in reducing neonatal mortality [27–29]. Although FLWs are known to face gender inequities in the course of their work [30], research is lacking on gender inequity among the recipients of FLW services. Such work is particularly needed because a role of such workers is to increase the demand for utilization of essential health services.

The aim of this article is to investigate whether sex of the infant is associated with maternal reports of infant health, health care use, and postnatal home visits from frontline health workers (FLWs) using data from a 2014 statewide representative household survey in Bihar, India, a large state (population 104 million) with high infant mortality (55 per 1000 live births) and sex ratio imbalance (918 females per 1000 males) [31,32]. Considerations of interactions of sex of the child with wealth and birth order are also explored. This work can offer insights into whether and to what degree gender inequities in infant health care seeking persist in Bihar, with the goal of guiding practical and policy solutions on how to address the excess female infant mortality [33] and sex ratio imbalance in India [31].

## METHODS

Data for this study were collected in March to June 2014 as the follow–up household survey for evaluation of Ananya, a public health program that supported a combination of supply–side and demand generation efforts to increase maternal and child health care utilization via the public health system in Bihar [34]. Evaluation of Ananya involved a two–armed quasi–experimental design, comparing mothers of infants and children from the eight districts in which the Ananya program was implemented to those from the remaining 30 districts assigned to the standard of care control condition. Although Ananya included both a baseline and one–year follow–up household survey, only the latter assessed neonatal health and health care utilization outcomes for mothers of 0– to 11–month–olds, providing variables for this analysis. Trained female study staff, subsequent to acquisition of written informed consent, collected all data. Details on the study’s multi–stage sampling and other procedures are available elsewhere [35].

The response rate for the sample of mothers of children 0 to 11 months in the survey was 87% (n = 11 654) [35]. The sample was further restricted for this analysis to mothers of living, singleton children (n = 11 570). Ethical approval for the original study was provided by India’s Health Ministry Screening Committee. Ethical approval for this analysis was provided by the University of California, San Diego.

### Measures

Dependent variables focused on health and curative and preventive health care utilization and were taken from India’s Demographic and Health Survey, where they had been validated with a comparable study population [36]. *Neonatal illness* was a binary measure determining whether the infant had any symptoms of illness in the first month of life, including loss of interest in breastfeeding, difficult/rapid breathing, feeling cold to touch, drowsy/difficult to arouse, yellowing of the skin or other symptoms noted by the mother. Neonatal illness was assessed in the subsample of mothers of children aged 1–11 months to allow for completion of the neonatal period (n = 10 836). *FLW advised seeking care* was a binary measure assessing whether FLWs had recommended seeking facility–based health care for the identified symptoms, and *received facility care* was a binary measure assessing whether the sick infant received care from a health worker (either public or private sector) for the identified symptoms; both were assessed only in the subsample of mothers of 1– to 11–month–olds reporting neonatal illness symptoms (n = 1217). *Hospitalization* since birth was a binary measure assessed for all mothers of infants aged 0–11 months with complete data (n = 11 557). Preventive health care use measures included *facility check–up at one month* (binary, assessed in the subsample of mothers of 0– to 11–month–olds with complete data n = 11 557), and *immunizations current* (defined as receipt of BCG and three Polio and DPT doses), assessed for infants 9–11 months old with complete data (n = 1994).

Measures related to FLW postnatal care home visits included *FLW visit within a week,* a binary measure assessed via a single item on whether such a visit occurred within one week of the index child’s birth (n = 11 556, all mothers with complete data). The quality of these visits was then assessed for the subsample of women who reported them (n = 1746) by asking if the visit included each of the following binary measures: *FLW discussed baby danger signs*, *FLW discussed exclusive breastfeeding*, *FLW discussed KMC* (kangaroo mother care, or exclusive breastfeeding with skin–to–skin contact), and *FLW discussed how to keep baby warm*.

The independent variable was sex of the infant. Covariates included: maternal age (categorized categorized in 5–year increments: 15–19, 20–24, 25–29, 30–34, or as 35 or older for the oldest age category, mother’s education (any vs none), urban/rural residence, birth order of index child (1st/2nd/3rd/4th or higher), whether or not the child had a living brother, and whether or not the participant resided in an Ananya focus district. Minority status (scheduled tribe/scheduled caste or Muslim) and wealth index were also included as covariates. In India, historically disadvantaged castes (Scheduled Castes – SC) and indigenous people (Scheduled Tribes – ST), have been recognized by the government as disadvantaged since independence and are accorded special protections and reservations. National data consistently document greater social, economic, and health disparities disadvantaging SC/ST individuals as well as Muslims, India’s largest religious minority group [4]. Household wealth index was constructed using principle component analysis using participants’ housing and asset information, including house construction materials, drinking water source, toilet type, cooking fuel, household members per sleeping room, electricity supply and household assets (such as television, bicycle, radio, car, mobile phone and others), with coefficients based on a prior year survey. This index was created in quartiles based on the larger study which included mothers of 0– to 23–month–olds; this study was restricted to 0– to 11–month–olds as comprehensive neonatal health data were only available for this subsample. Consequently, quartiles are not evenly distributed for this subsample.

### Data analysis

Multivariable logistic regression models adjusting for covariates were employed to investigate associations between sex of child and each study outcome. Covariates were considered based on social and other determinants of child health in India [14,37] and were included in models following the purposeful approach to model selection of Hosmer and Lemeshow [38]. Exploratory analyses also examined the interactions of child sex with wealth, birth order, minority community and mother’s age, to assess whether these covariates were potential modifiers of observed gender inequities, based on their observed importance in prior research [16,23,39]. Interactions were added separately to the adjusted model and defined as significant at *P* < 0.15. All analyses were conducted using the *svy* package in Stata SE 14 (Stata Corp, College Station, TX) and estimates make use of probability weights accounting for complex survey design and sampling.

## RESULTS

Female infants made up 46.3% (95% CI = 45.0–47.7) of the sample, which corresponds to an infant sex ratio of 863 females/1000 males (95% CI = 817–912) (**Table 1**). Crude logistic regression analyses indicate that a lower share of female infants had symptoms of neonatal illness compared to males (9.8% vs 11.9%; OR = 0.7, 95% CI = 0.6–0.9) and a lower share of female infants had been hospitalized compared to males (1.2% vs 4.9%; OR = 0.4, 95% CI = 0.3–0.6) (**Table 2**). However, compared to boys, girls had lower odds of receiving neonatal health care for identified symptoms (80.6% vs 89.1%; OR = 0.5, 95% CI = 0.3–0.8) and of receiving preventive care at a facility at 1 month (5.4% vs 7.3%; OR = 0.7, 95% CI = 0.6–0.9). Gender differences were not seen in immunizations, FLW postnatal visits or FLW advice to seek care. However, neither immunizations nor FLW visits occurred at target levels. Receipt of all immunizations (BCG, Polio 3 and DPT 3) by nine months was 59.9% for girls and 62.2% for boys. Only 16% of girls and boys had received a postnatal care home visit from an FLW in the first week post–partum. Multivariable analyses yielded similar association findings, with girls having lower odds than boys of having health concerns but also lower odds of receiving curative or preventive neonatal care (**Tables 3** and **4**). No effects of sex were seen on FLW visits.

**Table 1:** Sample characteristics (n=11 570)

Characteristic	Unweighted N	Weighted % (95% CI)*
**Gender of infant**		
Male	6231	53.7 (52.3–55.1)
Female	5339	46.3 (45.0–47.7)
**Infant’s age:**		
0–2 months	2864	25.0 (23.9–26.2)
3–5 months	3842	33.2 (32.1–34.5)
6–8 months	2862	24.5 (23.3–25.7)
9–11 months	2002	17.2 (16.2–18.2)
**Mother’s age:**		
15–19	443	4.2 (3.5–4.9)
20–24	4898	42.6 (41.1–44.2)
25–29	4302	35.8 (34.4–37.3)
30–34	1372	12.2 (11.3–13.1)
35+	555	5.2 (4.5–6.0)
**Wealth index:**		
Quartile 1 (Poorest)	3130	28.3 (26.3–30.4)
Quartile 2	2347	21.1 (19.7–22.5)
Quartile 3	2776	24.6 (23.2–26.1)
Quartile 4 (Richest)	3305	26.0 (24.1–27.9)
**Mother’s education:**		
No schooling	5860	52.3 (50.2–54.4)
Any schooling	5710	47.7 (45.6–49.8)
**Birth order:**		
1^st^ child	3625	31.2 (29.8–32.6)
2^nd^ child	3275	27.6 (26.4–28.9)
3^rd^ child	2274	20.1 (19.0–21.1)
4^th^ child or higher	2396	21.2 (19.9–22.5)
**Minority community designation:**		
Scheduled Caste/Scheduled Tribe	2979	26.1 (23.7–28.7)
Muslim	2026	17.3 (14.6–20.3)
Neither	6565	56.6 (53.7–59.5)
**Area of residence:**		
Urban	2135	10.3 (8.5–12.5)
Rural	9435	89.7 (87.5–91.5)
**Ananya District:**		
Yes	3072	24.2 (21.4–27.2)
No	8498	75.8 (72.8–78.6)
**Child has a living brother:**		
Yes	5135	44.4 (42.8–46.1)
No	6434	55.5 (53.9–57.2)

*Weighted % uses probability weights based on survey design and sampling. CI – confidence interval.

**Table 2:** Prevalence of illness, curative care, preventive care and frontline health worker measures by sex – bivariate analysis

Health or health care measure	Male prevalence		Female prevalence		Association of female sex with health measure	
**Weighted % (95% CI)**	**Unweighted n**	**Weighted %** **(95% CI)**	**Unweighted n**	OR (95% CI)*	***P***†
Symptoms of neonatal illness‡	12.9 (11.6–14.5)	723	9.8 (8.5–11.2)	494	0.7 (0.6–0.9)	0.001
FLW advised seeking care for neonatal illness§	16.8 (12.7–21.8)	113	19.0 (13.2–26.5)	84	1.2 (0.7–2.0)	0.58
Received care for neonatal illness§	89.1 (85.5–92.0)	634	80.6 (75.3–85.0)	397	0.5 (0.3–0.8)	0.003
Hospitalization	4.9 (4.1–5.8)	283	2.2 (1.8–2.8)	139	0.4 (0.3–0.6)	<0.001
Facility checkup at one month	7.3 (6.2–8.6)	465	5.4 (4.5–6.6)	311	0.7 (0.6–0.9)	0.01
Immunizations current at 9 months‖	62.2 (57.6–66.2)	677	59.9 (55.1–64.5)	59	0.9 (0.7–1.2)	0.52
FLW postnatal visit within a week	16.3 (14.6–18.2)	940	16.2 (14.6–18.0)	805	1 (0.9–1.2)	0.95
FLW discussed baby danger signs	30.8 (26.6–35.3)	281	30.0 (24.7–35.8)	261	1 (0.7–1.3)	0.80
FLW discussed exclusive breastfeeding	81.3 (77.2–84.7)	763	78.1 (72.7–82.8)	638	0.8 (0.6–1.2)	0.32
FLW discussed KMC	41.5 (36.8–46.5)	396	40.3 (34.9–46.0)	333	1 (0.7–1.2)	0.71
FLW discussed how to keep baby warm	39.3 (34.0–44.9)	371	37.3 (32.1–42.9)	315	0.9 (0.7–1.3)	0.63

CI – confidence interval, OR – odds ratio, FLW – frontline health worker, including community health workers (ASHAs), auxiliary nurse midwives, and social workers for children (Anganwadis), KMC – Kangaroo Mother Care

*Odds ratios are for females relative to males from simple logistic regression of the specified health/health care measure on sex

†All *P* values and 95% confidence intervals reflect Wald tests from simple logistic regression using probability weights based on survey design and sampling

‡Subsample of postneonatal infants (≥1 to 11 months old).

§For subsample of post–neonates who had experienced neonatal illness.

‖Subsample ≥9 months old; Immunizations current defined as having received Bacille Calmette–Guérin (BCG), 3 doses polio and 3 doses DPT (Diphtheria, Pertussis and Tetanus).

**Table 3:** Associations between sex of the child and illness, curative care and preventive care use – multivariable models

	Neonatal illness (n=10836)*		FLW advised seeking care for neonatal illness (n=1217)**†**		Received facility care for neonatal illness (n=1217)**†**		Hospitalization (n=11557)		Facility checkup at 1 month (n=11557)		Immunizations current at 9 months (n=1994)**‡**	
**Characteristic**	**aOR (95% CI)**	***P***§	**aOR (95% CI)**	***P***	**aOR (95% CI)**	***P***	**aOR (95% CI)**	***P***	**aOR (95% CI)**	***P***	**aOR (95% CI)**	***P***
**Female sex** vs. male	0.7 (0.6–0.9)	0.001	1.2 (0.7–2.0)	0.52	0.5 (0.3–0.8)	0.004	0.4 (0.3–0.6)	<0.001	0.7 (0.6–0.9)	0.02	0.9 (0.7–1.2)	0.52
**Mother’s age:**												
15–19	2.2 (1.1–4.2)	0.02	1.1 (0.2–7.5)	0.90	1.4 (0.3–6.7)	0.71	1.7 (0.6–4.5)	0.32	0.8 (0.4–1.6)	0.56	0.7 (0.3–1.8)	0.47
20–24	1.4 (0.8–2.3)	0.21	1.1 (0.3–3.9)	0.94	0.6 (0.2–2.1)	0.44	1.1 (0.5–2.3)	0.82	0.8 (0.5–1.4)	0.50	1 (0.5–1.9)	>0.99
25–29	1.1 (0.7–1.7)	0.71	1.1 (0.3–3.7)	0.93	0.7 (0.2–2.1)	0.49	1.2 (0.6–2.4)	0.58	0.8 (0.5–1.4)	0.45	0.8 (0.5–1.5)	0.55
30–34	1.3 (0.8–2.1)	0.30	0.8 (0.2–2.8)	0.69	0.9 (0.3–3.2)	0.90	1.2 (0.6–2.5)	0.66	1 (0.6–1.8)	0.87	0.8 (0.4–1.6)	0.59
35+	1 [Ref.]		1 [Ref.]		1 [Ref.]		1 [Ref.]		1 [Ref.]		1 [Ref.]	
**Wealth index:**												
Quartile 1 (Poorest)	0.9 (0.7–1.3)	0.63	0.4 (0.2–0.8)	0.01	0.5 (0.3–1)	0.04	0.4 (0.2–0.7)	0.001	0.7 (0.5–1.1)	0.10	0.6 (0.4–0.9)	0.02
Quartile 2	1.1 (0.8–1.5)	0.68	0.6 (0.2–1.3)	0.17	0.6 (0.3–1.2)	0.16	1 (0.6–1.5)	0.89	1 (0.7–1.5)	0.93	0.8 (0.5–1.2)	0.24
Quartile 3	1.0 (0.8–1.2)	0.69	0.8 (0.4–1.6)	0.51	0.8 (0.5–1.4)	0.49	0.9 (0.6–1.3)	0.47	0.9 (0.7–1.2)	0.54	0.8 (0.6–1.2)	0.28
Quartile 4 (Richest)	1 [Ref.]		1 [Ref.]		1 [Ref.]		1 [Ref.]		1 [Ref.]		1 [Ref.]	
**Mother’s education:**												
No schooling	1 [Ref.]		1 [Ref.]		1 [Ref.]		1 [Ref.]		1 [Ref.]		1 [Ref.]	
Any schooling	1.2 (0.9–1.5)	0.14	0.8 (0.5–1.3)	0.37	1.4 (0.9–2.2)	0.11	1.5 (1.0–2.1)	0.03	1.5 (1.2–2.0)	0.002	0.9 (0.7–1.2)	0.49
**Birth order:**												
4^th^ child or higher	1.1 (0.8–1.6)	0.62	0.7 (0.3–1.8)	0.45	0.9 (0.4–2.1)	0.83	1.2 (0.6–2.2)	0.57	0.7 (0.4–1.1)	0.12	1 (0.6–1.7)	0.96
3^rd^ child	1.0 (0.7–1.4)	0.95	0.9 (0.4–2.1)	0.88	1.1 (0.5–2.7)	0.81	0.8 (0.5–1.4)	0.50	0.7 (0.5–1)	0.05	1.3 (0.8–2)	0.27
2^nd^ child	0.8 (0.6–1.1)	0.10	0.8 (0.4–1.7)	.61	1.3 (0.7–2.5)	0.41	1 (0.7–1.4)	0.92	0.9 (0.6–1.2)	0.34	1 (0.7–1.4)	0.97
1^st^ child	1 [Ref.]		1 [Ref.]		1 [Ref.]		1 [Ref.]		1 [Ref.]		1 [Ref.]	
**Minority community:**												
Scheduled Caste/Scheduled Tribe	1 (0.8–1.3)	0.79	1.7 (1.1–2.9)	0.03	1.1 (0.6–1.8)	0.77	1.1 (0.8–1.5)	0.68	0.9 (0.7–1.2)	0.36	0.8 (0.6–1.2)	0.31
Muslim	1.4 (1.1–1.9)	0.02	0.4 (0.2–0.8)	0.01	1.3 (0.7–2.4)	0.34	1 (0.7–1.4)	0.90	0.9 (0.7–1.3)	0.63	0.7 (0.5–1)	0.03
Neither	1 [Ref.]		1 [Ref.]		1 [Ref.]		1 [Ref.]		1 [Ref.]		1 [Ref.]	
**Area of residence:**												
Urban	1 [Ref.]		1 [Ref.]		1 [Ref.]		1 [Ref.]		1 [Ref.]		1 [Ref.]	
Rural	1.1 (0.8–1.4)	0.58	2.3 (1.2–4.4)	0.01	1 (0.5–1.8)	0.88	1.5 (1–2.1)	0.04	0.8 (0.6–1)	0.10	1.1 (0.9–1.5)	0.40
**Ananya District:**												
Yes	1.2 (1.0–1.5)	0.05	1.3 (0.7–2.2)	0.39	0.7 (0.4–1)	0.05	1.5 (1.1–2.1)	0.01	2.2 (1.6–2.9)	<0.001	0.9 (0.7–1.2)	0.48
No	1 [Ref.]		1 [Ref.]		1 [Ref.]		1 [Ref.]		1 [Ref.]		1 [Ref.]	
**Child has a living brother:**												
Yes	1.1 (0.8–1.4)	0.57	1.2 (0.7–2.1)	0.59	1.1 (0.6–2.0)	0.73	0.6 (0.4–0.9)	0.01	0.8 (0.7–1.1)	0.14	1 (0.7–1.4)	0.91
No	1 [Ref.]		1 [Ref.]		1 [Ref.]		1 [Ref.]		1 [Ref.]		1 [Ref.]	

aOR – adjusted odds ratio, CI – confidence interval, Ref. – reference, FLW – frontline health worker, including community health workers (ASHAs), auxiliary nurse midwives, and social workers for children (Anganwadis).

*Subsample of postneonatal infants (≥1 month old).

†Subsample of post–neonates who had experienced symptoms of illness during the neonatal period.

‡≥9 months old. Immunizations current defined as having received Bacille Calmette–Guérin (BCG), 3 doses polio and 3 doses DPT (Diphtheria, Pertussis and Tetanus).

§All *P* values and 95% confidence intervals reflect Wald tests from multivariable logistic regression using probability weights based on survey design and sampling.

**Table 4:** Associations between sex of the child and frontline health worker postnatal home visits – multivariable models

	FLW visited within a week postpartum (n=11 556)		FLW discussed baby danger signs (n=1746)*		FLW discussed exclusive breastfeeding (n=1746)*		FLW discussed Kangaroo Mother Care (n=1746)*		FLW discussed how to keep baby warm (n=1746)*	
**Characteristic**	**aOR (95% CI)**	***P***†	**aOR (95% CI)**	***P***	**aOR (95% CI)**	***P***	**aOR (95% CI)**	***P***	**aOR (95% CI)**	***P***
**Female sex** vs. male	1.0 (0.9–1.2)	0.96	1.0 (0.7–1.4)	0.98	0.8 (0.5–1.2)	0.22	1.0 (0.7–1.2)	0.69	0.9 (0.7–1.3)	0.69
**Mother’s age:**										
15–19	2.1 (1.1–3.8)	0.02	1.6 (0.5–5.2)	0.40	0.4 (0.1–1.3)	0.12	1.5 (0.5–4.1)	0.44	1.5 (0.5–4.4)	0.47
20–24	1.4 (0.9–2.1)	0.18	0.8 (0.4–1.9)	0.63	0.5 (0.2–1.1)	0.09	1.1 (0.6–2.2)	0.78	1 (0.5–2.2)	0.96
25–29	1.3 (0.9–2.0)	0.19	1.6 (0.8–3.3)	0.21	0.8 (0.4–2.0)	0.68	2.3 (1.2–4.3)	0.01	1.5 (0.8–3.0)	0.25
30–34	1.4 (0.9–2.1)	0.11	1.9 (0.9–4.4)	0.11	0.9 (0.4–2.2)	0.83	2.5 (1.2–5.1)	0.01	1.3 (0.6–2.9)	0.47
35+	1 [Ref.]		1 [Ref.]		1 [Ref.]		1 [Ref.]		1 [Ref.]	
**Wealth index:**										
Quartile 1 (Poorest)	0.9 (0.7–1.1)	0.29	0.6 (0.4–1.0)	0.05	0.5 (0.3–0.9)	0.03	0.7 (0.5–1.1)	0.17	0.7 (0.4–1.1)	0.12
Quartile 2	1 (0.8–1.3)	0.92	1 (0.7–1.6)	0.85	1.2 (0.7–2.3)	0.52	1 (0.6–1.6)	0.94	1.2 (0.8–1.8)	0.37
Quartile 3	1.2 (0.9–1.4)	0.18	0.9 (0.5–1.5)	0.66	0.6 (0.4–1.0)	0.04	0.9 (0.6–1.4)	0.80	0.8 (0.5–1.4)	0.47
Quartile 4 (Richest)	1 [Ref.]		1 [Ref.]		1 [Ref.]		1 [Ref.]		1 [Ref.]	
**Any education** vs. no education	1.1 (0.9–1.3)	0.50	1 (0.7–1.6)	0.93	1.3 (0.8–2.1)	0.22	1.1 (0.8–1.6)	0.48	1 (0.7–1.4)	0.89
**Birth order:**										
4^th^ child or higher	1.2 (0.8–1.7)	0.35	0.4 (0.2–0.7)	0.001	0.9 (0.5–1.9)	0.86	0.5 (0.3–0.8)	0.009	0.8 (0.5–1.4)	0.41
3^rd^ child	0.9 (0.7–1.2)	0.57	0.3 (0.2–0.6)	0.001	0.9 (0.5–1.8)	0.80	0.7 (0.4–1.0)	0.08	0.8 (0.5–1.4)	0.48
2^nd^ child	0.9 (0.7–1.2)	0.45	0.7 (0.4–1.1)	0.11	0.9 (0.5–1.5)	0.69	0.6 (0.4–0.9)	0.02	1 (0.6–1.5)	0.88
1^st^ child	1 [Ref.]		1 [Ref.]		1 [Ref.]		1 [Ref.]		1 [Ref.]	
**Minority community:**										
Scheduled Caste/Scheduled Tribe	1.1 (0.9–1.4)	0.32	1.4 (0.9–2.1)	0.14	1.2 (0.7–1.8)	0.54	1.1 (0.8–1.6)	0.57	1.1 (0.7–1.5)	0.76
Muslim	0.9 (0.7–1.2)	0.38	1.2 (0.8–1.8)	0.33	1.2 (0.7–2.0)	0.43	1 (0.7–1.6)	0.84	1.2 (0.8–1.8)	0.32
Neither	1 [Ref.]		1 [Ref.]		1 [Ref.]		1 [Ref.]		1 [Ref.]	
**Area of residence:**										
Urban	1 [Ref.]		1 [Ref.]		1 [Ref.]		1 [Ref.]		1 [Ref.]	
Rural	2.3 (1.7–3.0)	0.001	0.6 (0.4–1.1)	0.11	0.9 (0.6–1.6)	0.78	1 (0.6–1.7)	0.96	0.6 (0.4–1.0)	0.04
**Ananya District:**										
Yes	1.2 (1.0–1.5)	0.12	1.1 (0.7–1.7)	0.63	1.3 (0.9–1.9)	0.18	1.4 (1.0–2.0)	0.06	1.5 (1.1–2.1)	0.02
No	1 [Ref.]		1 [Ref.]		1 [Ref.]		1 [Ref.]		1 [Ref.]	
**Child has a living brother:**										
Yes	1.2 (1.0–1.5)	0.03	1.2 (0.8–1.8)	0.34	1 (0.6–1.6)	0.93	1 (0.7–1.4)	0.98	1 (0.7–1.4)	0.93
No	1 [Ref.]		1 [Ref.]		1 [Ref.]		1 [Ref.]		1 [Ref.]	

aOR – adjusted odds ratio, CI – confidence interval, Ref. – reference, FLW: frontline health worker, including community health workers (ASHAs), auxiliary nurse midwives, and social workers for children (Anganwadis)

*Subsample reporting an FLW postnatal care visit in the first week postpartum.

†All *P* values and 95% confidence intervals reflect Wald tests from multivariate logistic regression using probability weights based on survey design and sampling.

Stratum–specific odds ratios for interactions between sex of child and birth order, as well as sex of child and wealth, are presented in **Table 5** for outcomes associated with child sex in the primary analyses and demonstrating significant interaction effects (*P* < 0.15). Only receipt of care for neonatal illness met these criteria. In the case of birth order modifying the effect of sex on receipt of care for neonatal illness, there was no difference among firstborn children but an increasing disparity for females with higher birth order. This culminated in girls having 90% reduced odds of receiving care relative to boys when restricting to fourth or higher child (adjusted OR (aOR) = 0.1, 95% CI = 0.1–0.3). Wealth index also demonstrated significant effect measure modification with child sex in the model predicting receipt of care for neonatal symptoms. The gender inequity disfavoring females was most pronounced among the poorest quartile; similarly, the disparity between highest and lowest quartiles of wealth was more pronounced among females than males. Among the lowest wealth quartile, girls’ odds of receiving care were 80% reduced relative to boys (aOR = 0.2, 95% CI = 0.1–0.5). Similarly, among females, those in quartile 1 had lower odds of this outcome relative to those in quartile 4 (OR = 0.3; 95% CI = 0.1–0.7); this association of wealth with receiving neonatal care did not hold among males.

**Table 5:** Stratum–specific associations between sex of child and receipt of care for neonatal illness*

Interaction stratum	Main effect	Received care for neonatal symptoms (n=1217)**†**	
**aOR (95% CI)**	***P***‡
**Child sex × birth order:**			**0.01**
4^th^ child or higher	Female vs. male	0.1 (0.1–0.3)	<0.001
3^rd^ child	Female vs. male	0.5 (0.2–1.2)	0.11
2^nd^ child	Female vs. male	0.6 (0.2–1.5)	0.27
1^st^ child	Female vs. male	1.0 (0.5–2.3)	0.92
			
Female	4^th^ or higher vs. 1^st^ child	0.4 (0.1–1.0)	0.05
	3^rd^ vs. 1^st^ child	0.7 (0.3–2.2)	0.59
	2^nd^ vs. 1^st^ child	1.0 (0.4–2.5)	0.94
Male	4^th^ or higher vs. 1^st^ child	2.6 (0.9–7.0)	0.07
	3^rd^ vs. 1^st^ child	1.6 (0.6–4.4)	0.35
	2^nd^ vs. 1^st^ child	1.7 (0.8–3.8)	0.2
			
**Child sex × wealth index:**			**0.06**
Quartile 1 (Poorest)	Female vs. male	0.2 (0.1–0.5)	<0.001
Quartile 2	Female vs. male	1.2 (0.5–3.0)	0.75
Quartile 3	Female vs. male	0.6 (0.3–1.4)	0.25
Quartile 4 (Richest)	Female vs. male	0.6 (0.3–1.4)	0.25
			
Female	Quartile 1 vs. 4	0.3 (0.1–0.7)	0.005
	Quartile 2 vs. 4	0.9 (0.4–2.2)	0.75
	Quartile 3 vs. 4	0.8 (0.3–1.8)	0.58
Male	Quartile 1 vs. 4	0.9 (0.4–2.2)	0.85
	Quartile 2 vs. 4	0.5 (0.2–1.1)	0.08
	Quartile 3 vs. 4	0.8 (0.4–1.7)	0.57

aOR – adjusted odds ratio, CI – confidence interval

*In addition to the interaction and main effects, the interaction models included remaining covariates of maternal age, household wealth index, mother’s education, birth order of index child, minority community (caste or religion), rural residence, whether or not the participant resided in an Ananya focus district, and whether or not the child had a living brother. Logistic regression models based on the two interactions displayed, birth order and wealth, were tested with an overall Wald test and found to be significant at alpha=0.15. Odds ratios within strata based on those interactions are displayed.

†Subsample of post–neonates who had experienced neonatal illness.

‡All p–values and 95% confidence intervals are from logistic regressions using probability weights based on survey design and sampling.

## DISCUSSION

Results of this study document significant gender inequities in indicators of curative and preventive neonatal health care use, with girls having lower odds than boys to receive care for neonatal illness and facility–delivered postnatal wellness care. Moreover, observed gender inequity in neonatal care seeking is more pronounced among infants with a larger number of siblings and among households in the poorest wealth quartile, conditions likely associated with a relative scarcity of household resources. These findings indicate that, as seen in smaller scale and older studies from India [18,21,24], gender bias continues to affect parents’ health care seeking for the child, and these effects are exacerbated when household resources are limited or over–extended. FLW visits, although currently at low numbers, encouragingly do not exhibit the same bias.

Despite the greater odds of non–receipt of care, girls had lower odds of being reported ill during the neonatal period compared to boys. This finding corresponds with national indications of lower risk of neonatal death for girls relative to boys, suggesting greater biologic vulnerability for boys relative to girls [9]. The odds of hospitalization in the first year of life, a more rare and expensive event, differed even more starkly between boy and girl infants, and as with neonatal illness, may be attributable to biology. However, it may also, at least in part, be attributable to greater use of hospitalization when needed for boys relative to girls. As noted previously, research from India documents that higher–cost medical care, such as hospitalization, is more likely to be prioritized and used when needed for boys relative to girls [18,24,39], while discharge against medical advice is more likely for girls relative to boys [21].

Importantly, immunization coverage did not differ by infant sex at 9 months, a finding contrary to a number of studies conducted in India in the past [13–16]. These findings suggest that the growing efforts to maximize immunization coverage since 2006–2007 – when much of the data on gender disparities in immunization were collected – are not only expanding coverage but also reducing gender inequities [40]. Of note, these improvements were based on access to care broadly and not on addressing underlying causes of gender inequities in immunization uptake. Consequently, ongoing gender inequities in use of other health services persist, suggesting that improving access, while important, will be inadequate to address observed gender inequities in care. More work is needed to identify how to work more effectively with parents to increase their value for their girl children, particularly in the presence of older children and male children. Reinforcing this point is a recent analysis of infant and child mortality in neighboring Odisha state, which indicated that while infant and child mortality significantly decreased from 2006 to 2012, the trend was not observed for girls [41].

A related paradox of extending health programs to combat social inequalities in health is that boys have greater access to these facility–based services than do girls [33]. Use of FLWs via home visits may provide an opportunity to extend reach to girls more effectively, particularly for those from more vulnerable impoverished households [26]. Study findings suggest no gender bias in FLWs’ provision of a postnatal visit or advice for child health care seeking. Unfortunately, too few women received a FLW postnatal visit, only 16%. This is particularly disappointing given the promise FLWs offer to help address parents’ gender bias in care seeking. A post hoc analysis from these data showed a non–significant trend (*P* = 0.09) indicating that girl infants who were advised to seek care by an FLW had a higher odds of having received care compared to girls not so advised (89.4% vs 78.6%; OR = 2.3, 95% CI = 0.9–6.2). This association did not hold among boys, who had higher overall levels of care seeking. Together, these findings suggest that improving coverage of FLW services could play a role in alleviating the gender inequity in care–seeking for infants alongside efforts to improve standing of the girl child.

In addition to findings related to gender inequities and health, some findings regarding social inequities were also observed. Adolescent and Muslim mothers had higher odds of having a sick neonate, and among those with a sick neonate, Muslims had lower odds of having a front line worker (FLW) recommend that they seek care for their infant. Interestingly, there was a higher odds of FLWs recommending care for sick infants more to SC/ST and rural mothers, suggesting that FLWs do support care for more socially vulnerable groups, but perhaps not evenly. Notably, the poorest infants appear to be the most vulnerable, having lower odds than the richest infants of having a FLW recommend care when ill, to receive care when ill, to be hospitalized, and to receive vaccinations, suggesting that poverty remains a key driver for non–use of health care in Bihar. These findings may help explain the heightened risk for infant mortality among the abject poor in India [42]. Sadly, and as noted above, poverty appears to exacerbate what appears to be gender discriminatory effects on neonatal care utilization.

Some study limitations must be considered. Analyses are cross–sectional, so causality cannot be assumed. The data are self–reported by mothers and thus subject to recall and social desirability biases. Additionally, mortality data were not available for this analysis. Participation biases may exist, and we unfortunately did not collect data on those who declined participation to ascertain what these biases may be; the high participation rate (87%, as noted in the methods) reduces our vulnerability to these potential biases. A key challenge for interpretation of this study and many that investigate gender bias in health care is that some of the important outcomes represent both health need and health care utilization. Differentials in hospitalization of baby boys and girls likely reflect differences in severity of underlying illness, gender bias in parents’ use of care or referral systems’ recommendations for care. Bhan and colleagues found hospitalization for diarrheal illness in a New Delhi hospital was much lower among girls, but mortality was higher at the same time, undermining differential vulnerability as an explanation [23]. Additionally, symptoms of illness in the first month can reflect perception or actual rates of illness [13]. In our survey, the key question about actual receipt of care, restricted to those who endorsed neonatal illness, shows a marked gender inequity, as does the preventive care outcome of one–month well visits at a facility. These together suggest gender inequity even within the recognized and reported cases of illness and further imply that the same underlying gender bias could contribute to other findings such as the stark difference in hospitalizations. Assessing the presumed effect of health care use on mortality with any possible effect modification by gender would be an important future direction for this research.

## CONCLUSION

This study documents significant gender inequities disadvantaging infant girls in the receipt of facility–based curative and preventive health care in Bihar, India, implying a role of gender bias and neglect on the part of parents. Notably, gender inequity in immunization coverage at 9 months of age was not observed, suggesting dramatic improvement on this issue over the past decade [13–17], likely via expansion in local access to vaccinations [40]. Front–line worker visits and services were not implicated in the gender disparity. These findings suggest that focus on gender bias and its impact on parents’ health care decision–making remains a concern in Bihar, and inadequate use of health services for girls may be contributing to the excess female mortality rate in the country. Study findings indicate that poverty and a higher number of siblings worsened observed gender inequities and suggest a role for targeting those particularly limited by resources. Broader reach of care beyond the facility, possibly through FLWs and home visits, may offer opportunity to support improvement, but such efforts must coincide with broader social change to support the value of the girl child in India and parents’ recognition of this value.

## References

[1] Park JJ, Brondi L (2015). Why are girls still dying unnecessarily? The need to address gender inequity in child health in the post-2015 development agenda.. J Glob Health.

[2] Ostrowska A (2012). Health inequalities–gender perspective.. Przegl Lek.

[3] Grown C, Gupta GR, Pande R (2005). Taking action to improve women’s health through gender equality and women’s empowerment.. Lancet.

[4] IIPS. NFHS-4 Fact Sheets for Key Indicators Based on Final Data. June 2017. http://rchiips.org/nfhs/factsheet_NFHS-4.shtml (accessed February 2, 2016.

[5] UNICEF. UNICEF global databases, 2016, based on DHS, MICS and other nationally representative surveys. Feb 2017. https://data.unicef.org/topic/child-protection/child-marriage/#.

[6] Devries KM, Mak JY, Garcia-Moreno C, Petzold M, Child JC, Falder G (2013). Global health. The global prevalence of intimate partner violence against women.. Science.

[7] Kishoor S, Gupta K. Gender Equality and Women’s Empowerment in India: National Family and Health Survey 3 India. International Institute of Population Sciences; Deonar, Mumbai: Ministry of Health and Family Welfare, Government of India, 2009.

[8] Jha P, Kesler MA, Kumar R (2011). Trends in selective abortions of girls in India: analysis of nationally representative birth histories from 1990 to 2005 and census data from 1991 to 2011.. Lancet.

[9] Alkema L, Chao F, You D, Pedersen J, Sawyer CC (2014). National, regional, and global sex ratios of infant, child, and under-5 mortality and identification of countries with outlying ratios: a systematic assessment.. Lancet Glob Health.

[10] Requejo J, Victora C, Bryce J. A Decade of Tracking Progress for Maternal, Newborn and Child Survival. The 2015 Report., 2015. 10.1016/S0140-6736(15)00519-X PMC7613171 26477328

[11] Gupta R, Makhija S, Sood S, Devgan V (2016). Discrimination in Seeking Medical Care for Female Child from Birth to Adolescence - A Retrospective Study.. Indian J Pediatr.

[12] Nair H, Campbell H, Park JJ, et al. Common childhood infections and gender inequalities: a systematic review. UNICEF Maternal, Newborn and Child Health Working Papers. New York, NY; 2015.

[13] Corsi DJ, Bassani DG, Kumar R (2009). Gender inequity and age-appropriate immunization coverage in India from 1992 to 2006.. BMC Int Health Hum Rights.

[14] Prusty RK, Kumar A (2014). Socioeconomic dynamics of gender disparity in childhood immunization in India, 1992-2006.. PLoS One.

[15] Singh PK, Parasuraman S (2014). ‘Looking beyond the male-female dichotomy’ - sibling composition and child immunization in India, 1992-2006.. Soc Sci Med.

[16] Pande RP (2003). Selective gender differences in childhood nutrition and immunization in rural India: the role of siblings.. Demography.

[17] Singh A (2012). Gender based within-household inequality in childhood immunization in India: changes over time and across regions.. PLoS One.

[18] Willis JR, Kumar V, Mohanty S (2009). Gender differences in perception and care-seeking for illness of newborns in rural Uttar Pradesh, India.. J Health Popul Nutr.

[19] Shah R, Mullany LC, Darmstadt GL (2014). Determinants and pattern of care seeking for preterm newborns in a rural Bangladeshi cohort.. BMC Health Serv Res.

[20] Thind A (2004). Health Service Use by Children in Rural Bihar.. J Trop Pediatr.

[21] Kshirsagar V, Ahmed M, Colaco S (2013). A study of gender differences in treatment of critically ill newborns in NICU of Krishna hospital, Karad, Maharashtra. Natl J Community Med.

[22] Saha A, Parmar V, Chawala D, Walia D (2009). Gender bias in utilization of health services in Chandigarh.. Indian J Pediatr.

[23] Bhan G, Bhandari N, Taneja S, Mazumder S, Bahl R (2005). The effect of maternal education on gender bias in care-seeking for common childhood illnesses..

[24] Pandey A, Sengupta G, Mondal SK (2002). Gender differences in healthcare-seeking during common illnesses in a rural community of West Bengal, India.. J Health Popul Nutr.

[25] Das Gupta M (1987). Selective discrimination against female children in rural Punjab, India.. Popul Dev Rev.

[26] McCollum R, Gomez W, Theobald S, Taegtmeyer M (2016). How equitable are community health worker programmes and which programme features influence equity of community health worker services? A systematic review.. BMC Public Health.

[27] Gogia S, Sachdev HP (2016). Home-based neonatal care by community health workers for preventing mortality in neonates in low- and middle-income countries: a systematic review.. J Perinatol.

[28] Mangham-Jefferies L, Pitt C, Cousens S, Mills A, Schellenberg J (2014). Cost-effectiveness of strategies to improve the utilization and provision of maternal and newborn health care in low-income and lower-middle-income countries: a systematic review.. BMC Pregnancy Childbirth.

[29] Gilmore B, McAuliffe E (2013). Effectiveness of community health workers delivering preventive interventions for maternal and child health in low- and middle-income countries: a systematic review.. BMC Public Health.

[30] George A. (2008). Nurses, community health workers, and home carers: gendered human resources compensating for skewed health systems.. Glob Public Health.

[31] Raj A, Kidd JD, Cheng DM (2013). Associations between partner violence perpetration and history of STI among HIV-infected substance using men in Russia.. AIDS Care.

[32] Census of India. 2011. New Delhi: Office of the Registrar General & Census Commissioner, Government of India.

[33] Khera R, Jain S, Lodha R, Ramakrishnan S (2014). Gender bias in child care and child health: global patterns.. Arch Dis Child.

[34] Smith K, Rangarajan A, Borkum E, Dandona L. Measurement, Learning and Evaluation for the Ananya Program (Family Health Initiative in Bihar): Design Report: Mathematica Policy Research, 2011.

[35] Borkum E, Rotz D, Rangarajan A, et al. Midline Findings from the Evaluation of the Ananya Program in Bihar: Mathematica Policy Research, 2014.

[36] National Family Health Survey (NFHS-3), 2005–06: India: Volume I. Mumbai, IIPS: International Institute for Population Sciences (IIPS) and Macro International, 2007.

[37] Balarajan Y, Selvaraj S, Subramanian SV (2011). Health care and equity in India.. Lancet.

[38] Hosmer D, Lemeshow S, Sturdivant RX. Model-building strategies and methods for logistic regression. Applied logistic regression 3rd Edition. Hoboken, New Jersey: John Wiley & Sons, Inc.; 2013: 89–151.

[39] Asfaw A, Lamanna F, Klasen S (2010). Gender gap in parents’ financing strategy for hospitalization of their children: evidence from India.. Health Econ.

[40] WHO, UNICEF. Updated data on immunization coverage published by WHO and UNICEF. Geneva, Switzerland: World Health Organization; July 2015.

[41] Thomas D, Sarangi BL, Garg A (2015). Closing the health and nutrition gap in Odisha, India: A case study of how transforming the health system is achieving greater equity.. Soc Sci Med.

[42] Mohanty SK (2011). Multidimensional poverty and child survival in India.. PLoS One.

